# Developing an app-based self-management program for people living with HIV: a randomized controlled pilot study during the COVID-19 pandemic

**DOI:** 10.1038/s41598-022-19238-w

**Published:** 2022-11-12

**Authors:** Mi-So Shim, Sunah Kim, Mona Choi, Jun Yong Choi, Chang Gi Park, Gwang Suk Kim

**Affiliations:** 1 Department of Nursing, College of Nursing, Keimyung University, Deagu, Republic of Korea; 2 College of Nursing, Mo-Im Kim Nursing Research Institute, Yonsei University, 50-1 Yonsei-ro Seodaemun-gu, Seoul, 03722 Republic of Korea; 3 Department of Internal Medicine, College of Medicine, Yonsei University, Seoul, Republic of Korea; 4 Department of Population Nursing Science, College of Nursing, University of Illinois, Chicago, IL USA

**Keywords:** Infectious diseases, Health care

## Abstract

People living with human immunodeficiency virus (PLWH) in Korea demonstrate insufficient self-management behaviors. Especially during pandemics such as COVID-19, technology-based self-management programs are needed to overcome time and space limitations. The purpose of this study was to evaluate the effects of a self-management program using a mobile app (Health Manager) on self-management outcomes among PLWH in Korea. A randomized controlled pilot trial was performed and participants were enrolled in the infectious outpatient clinic of a single hospital. The intervention group used the mobile app for 4 weeks, while the control group received self-management education materials in a portable document format. The online self-report questionnaire assessed primary outcomes including self-efficacy for self-management, self-management behaviors, and medication adherence, and secondary outcomes including perceived health status, depression, and perceived stigma. Thirty-three participants were randomly assigned to the intervention (n = 17) or the control group (n = 16). In the intention-to-treat analysis, self-efficacy for self-management and self-management behaviors increased, while perceived stigma decreased. The app-based self-management program could be considered a helpful strategy to improve self-management outcomes among PLWH and reduce their perceived stigma during the pandemic. Further studies with larger samples and longer follow-ups are needed.

*Trial registration*: Clinical Research Information Service, KCT0004696 [04/02/2020].

## Introduction

Human immunodeficiency virus (HIV) infections, which are chronic diseases, can be managed using antiretroviral treatment (ART)^1^. Similar to other chronic illnesses, self-management is essential for people living with HIV (PLWH) to promote their physical and mental health, and maintain social relationships while undergoing treatment^2^; this can help them engage in regular lifestyles^3,4^ and prevent infection spread through sexual contact^4^. In Korea, there were 1,016 new HIV infections in 2020, with 78.5% occurring among adults aged between 20 and 40 years^5^. Self-management immediately after being diagnosed is essential for these relatively young PLWH to remain healthy and prevent further HIV transmission. However, PLWH in Korea have shown insufficient self-management behaviors. The proportion of PLWH who maintained medication adherence of 95% or more and were capable of suppressing viruses was 70.4%^6^. The results were better than those of some developed and developing countries (sub-Saharan African countries, North American countries, and France), but lower than the 90% target of the Joint United Nations Programme on HIV/AIDS^6^. Similarly, the National Human Rights Commission of Korea reported that PLWH have poorer levels of health indicators, such as smoking, drinking, stress, depression, and suicide attempts, than the general public^7^.

During the coronavirus disease-2019 (COVID-19) pandemic, PLWH were exposed to greater physical and psychological burdens^10,11^. Regarding Korea, since the first confirmed case of COVID-19 in January 2020, new cases have been increasing steadily^12^. As of January 4, 2020, the cumulative number of newly confirmed cases and the number of deaths due to COVID-19 were 645,226 and 5,781, respectively^12^. A previous review reported that COVID-19 symptoms were not severe in advanced-stage HIV patients^13^, however, the risk of hospitalization and mortality due to COVID-19 was higher in HIV patients than in non-infected patients^11^. In addition, physical distancing for the prevention of COVID-19 had a negative impact on health care access among PLWH and increased their social isolation and loneliness^10^.

Various obstacles also affect health care service access among PLWH. The stigma regarding having HIV, inadequate competence of health systems and healthcare providers (HCPs), and insufficient acceptance and emotional support from the HCPs are obstacles to treatment for PLWH^2,8^. McDonald et al. reported that doctors are the most important source of support for PLWH; however, time shortage and prejudice inhibits them from discussing self-management and using community resources to support PLWH^9^. Thus, many PLWH cannot access health promotion services and are unable to reveal their HIV status. Therefore, self-management interventions that use different methods to overcome the time and space limitations related to their implementation are necessary. Furthermore, mobile applications (apps) can provide services without such restrictions; they can be used as an alternative for PLWH, as stigma hinders them from participating in offline communities^14^.

A previous review reported that self-management interventions including self-management skills training and counseling were effective in improving the self-management outcomes of PLWH, such as with symptom management, medication adherence, and self-management behaviors^15^. In addition, technology-assisted self-management interventions using virtual video clips, text messages, and phone calls were effective for improving self-management outcomes^15^. Recent studies have also shown that mobile app-based interventions improved treatment retention, medication adherence, and health indicators such as CD4 + T cell count and blood viral load^14,16^, and mitigated major HIV symptoms such as anxiety, depression, neuralgia, fever, chills, and weight loss among PLWH^17^. Furthermore, it was helpful to reduce the stigma and discrimination against PLWH by providing enhanced social support, opportunities to disclose their infection status, and secure online spaces for participation without criticism^18^. However, in Korea, limited studies provide self-management interventions to improve self-efficacy or self-management among PLWH^19^. Further, a study evaluating the effectiveness of a self-management intervention using a mobile app can improve the comprehensive self-management of PLWH especially during pandemics such as COVID-19.

The primary aim of this study was to evaluate the effects of an app-based self-management program for PLWH on self-management outcomes such as self-efficacy for self-management, self-management behaviors, and medication adherence during the COVID-19 pandemic. This study also examined the effects of the program on perceived health status, depression, and perceived stigma of HIV among PLWH.

## Methods

### Study design

A randomized controlled pilot trial was conducted to evaluate the self-management program’s effectiveness. The study protocol was registered with the Clinical Research Information Service [Registration number KCT0004696, 04/02/2020].

### Participants

PLWH in Korea were recruited using convenient sampling. Participants were enrolled in the infectious outpatient clinic of a single hospital by the researcher. The inclusion criteria were: being diagnosed with HIV, aged above 19 years, receiving ART, using an Android smartphone, and being able to read and respond to questionnaires using a smartphone. Exclusion criteria were receiving a medical diagnosis that required immediate treatment and counseling in addition to HIV diagnosis. The required sample size was estimated using G*power 3.1 software^20^. The sample size derived was 28 when the effect size was 0.25, the first type error was 5%, the power was 80%, the number of comparison groups was two, and the number of measurements was three. Considering a drop out in the final sample, we divided 33 participants into intervention and control groups. No adverse events were reported by any of the participants.

The enrollment and follow-up were conducted from April to June 2020. The researcher randomly assigned the participants to the intervention and control groups in a 1:1 ratio via the Research Randomizer platform^21^. The intervention group employed the app for 4 weeks, while the control was provided with self-management educational materials in a portable document format. The nature of the intervention prevented participant blinding.

### Health manager mobile app intervention

**Figure 1:**
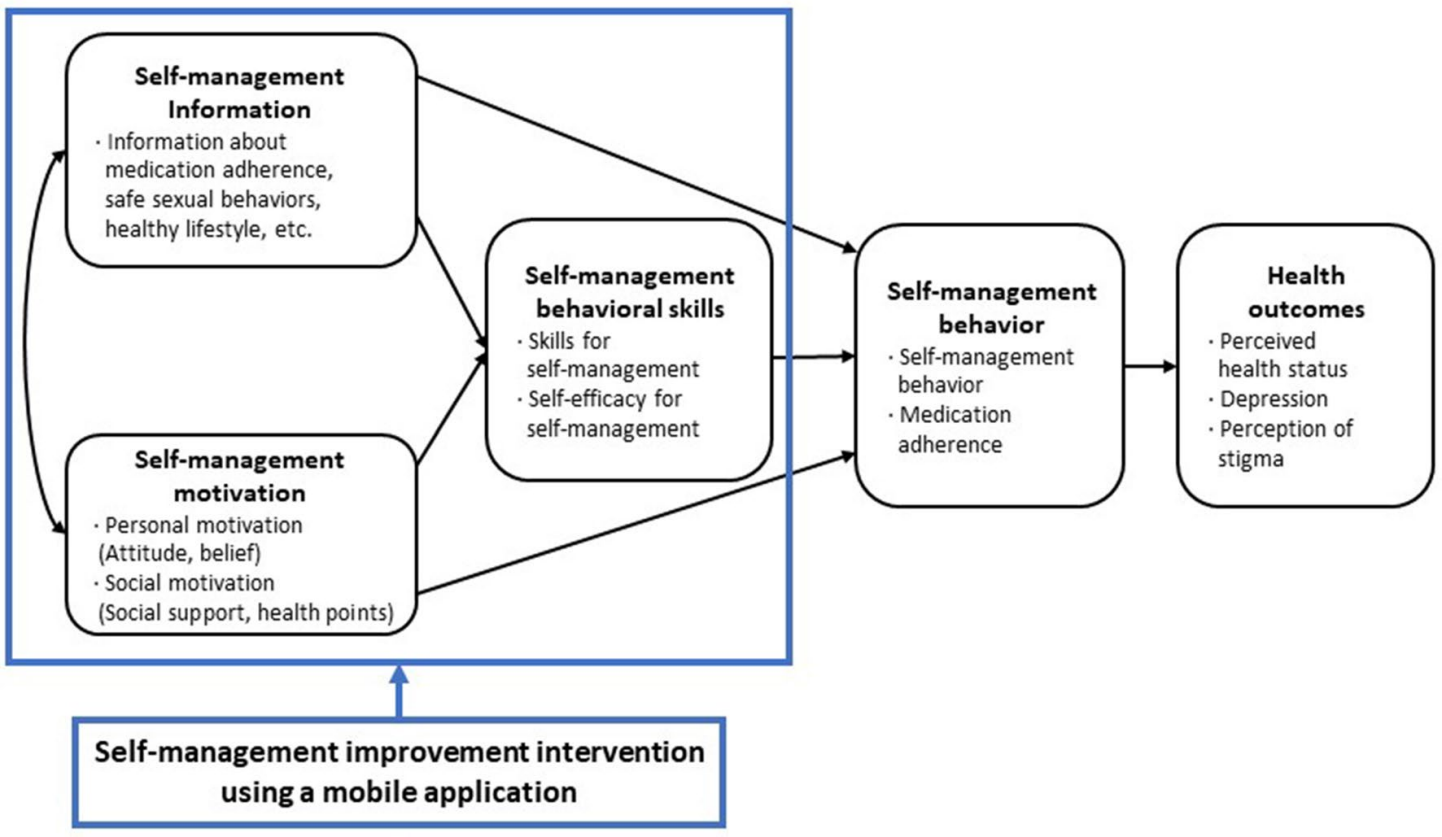
Conceptual framework of the self-management program using a mobile application.

**Figure 2:**
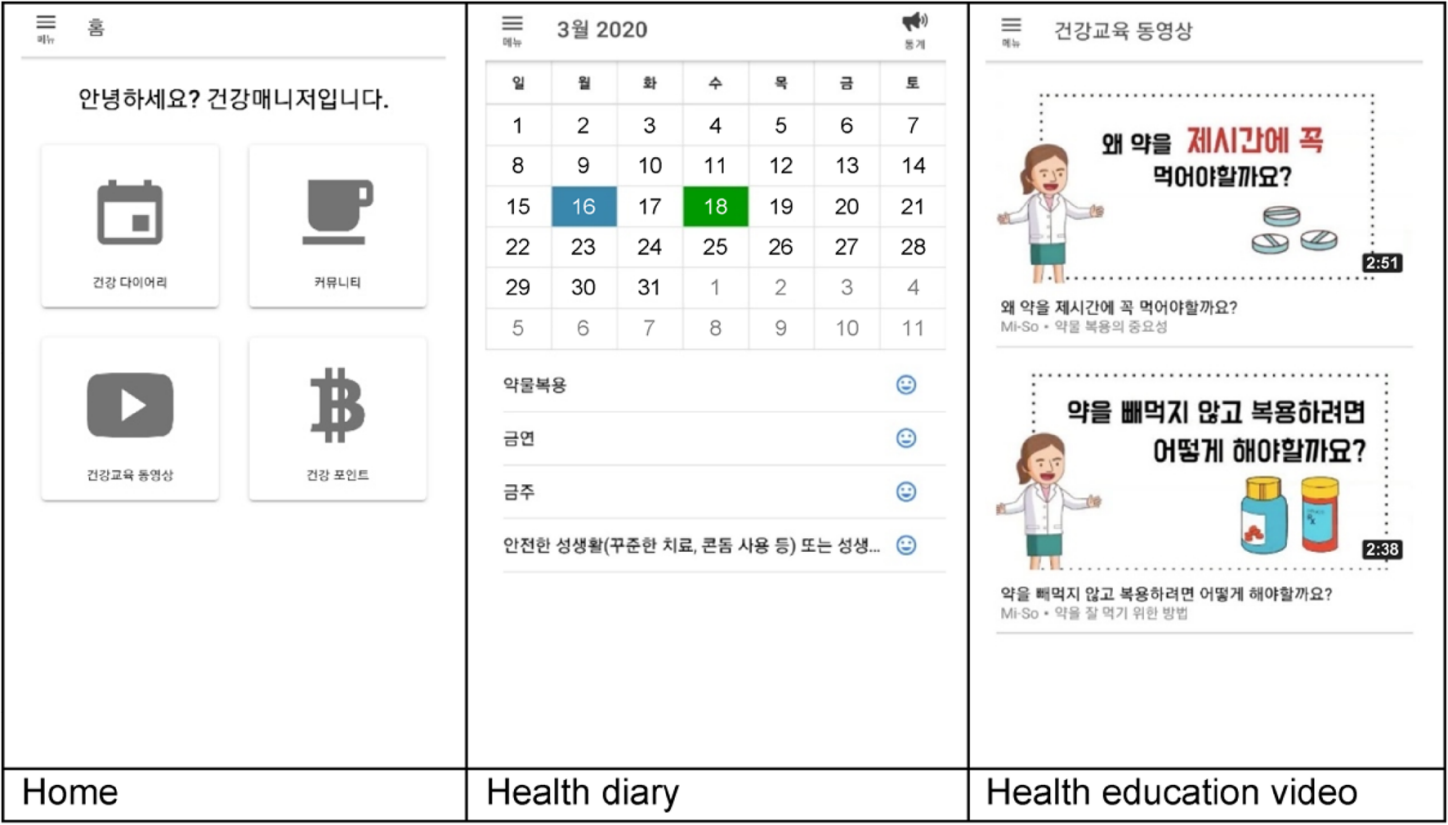
Screenshots of the mobile application’s main menu.

This intervention was developed based on the information-motivation-behavioral skills (IMB) model^22,23^ (Fig. 1). The “Health Manager” mobile app had 13 menus: Home, My Info, Health Goals, Alarms, Health Diary, Education Video, Health Information Bulletin Board, Community, Health Counselling, Health Points, Survey, Settings, and Help. The participants engaged in the self-management program through this app for 4 weeks (Fig. 2).

Intervention group participants installed the app on their mobile phones through the webpage as guided by the researcher. After user registration and the researcher’s approval process, they logged in to the mobile app. The participants were automatically shown the “Help” menu on the first login which informed them about the menus and the app’s usage. The last page of the “Help” menu was linked with the “Health goals” menu to enter the participants’ health information and goals for medication adherence, smoking, alcohol consumption, and sex life, and set a time for the medication alarm.

The self-management information was provided through the “Educational videos” and “Health information bulletin board” menus. Nine videos on medication adherence, smoking, alcohol consumption, and sex life were included, that could be watched per convenience; on the bulletin board, the researcher updated news about HIV and the health care information on mental health and strengthening immunity once or twice weekly. To improve self-management motivation, the “Community,” “Health counseling,” and "Health points” menus were used. The “Community” menu comprised three bulletin boards to share health care tips, worries, and their own stories freely with other PLWH. Participants could post at any time and communicate through replies. In the “Health counseling” menu, one-on-one counseling with nurses was provided through the bulletin board. Additionally, in the “Health points” menu, the participants could check the points accumulated through community activities (posts and replies), diary writing, and surveys, and exchange them for online gift vouchers. Through the “Health goals,” “Alarms,” and “Health diary” menus, the participants learned and employed behavioral skills for self-management by setting objectives regarding medication adherence, smoking, alcohol consumption, and sex life; setting reminders for medications (daily) and receiving encouragements to achieve their health goals (thrice a week); and recording their medication schedule and self-management behaviors.

### Measurement

#### Primary outcomes

The primary outcomes of this study were self-efficacy for self-management, self-management behaviors, and medication adherence, and these variables related to self-management outcomes. Self-efficacy for self-management was assessed using the eight items developed by Wallston et al., translated into Korean by the researchers with the authors’ permission^24^. Each item was rated on a six-point Likert scale (1–6); the number of points corresponded to the level of self-efficacy. Items one, two, six, and seven were calculated inversely. This scale’s Cronbach’s α was 0.78 and 0.65 in Wallston et al.’s study^24^ and this research, respectively.

Self-management behaviors of PLWH were assessed using Webel et al.’s^25^ scale, translated into Korean by Kim et al.^26^. Subsequently, Kim et al. added four questions^27^ after in-depth interviews with PLWH. Each item was rated on a four-point Likert scale (0–3); the number of points corresponds to the level of the self-management behaviors. This scale’s Cronbach’s α was 0.87 and 0.82 in Kim et al.’s study^27^ and this research, respectively.

The self-reported questionnaire about medication adherence, proposed by Simoni et al., was adapted for the Korean context to evaluate consultation services for PLWH in hospitals by the Korea Disease Control and Prevention Agency (KCDA)^28^. The original scale included 5 questions regarding forgetting to take medication, 24 regarding why medication was not taken, 1 about the rate at which the respondent had taken medication during the past month, and questions about CD4 + T cell counts and blood viral load. The questionnaire in this study had six questions: five regarding forgetting to take medication and one about the rate at which the respondent had properly taken medication during the past month; the latter item was used to analyze the intervention’s effect.

#### Secondary outcomes

The secondary outcomes of this study were perceived health status, depression, and perceived stigma of HIV. Perceived health status was measured using a five-point Likert scale (very bad, bad, moderate, good, and very good) for the question “What is your overall health status currently?”.

The Korean version of the Patient Health Questionnaire-9 developed by Kroenke et al. was used to measure depression^29^. This scale was provided for free by Pfizer. Each item was rated on a four-point Likert scale (0–3); the scores were correlated with the level of depression. This scale’s Cronbach’s α was 0.87 for PLWH in Choi et al.’s study^30^ and 0.93 in this research.

Perceived stigma regarding HIV was determined using six items from the scale developed by Kalichman et al.^31^. Positive and negative responses were assigned “1” and “0” points, respectively. The total score corresponded to the perceived degree of stigma. This scale’s Cronbach’s α was 0.75 and 0.73 in Kalichman et al.’s study^31^ and in this research, respectively.

#### Satisfaction with the program (intervention group only)

Program satisfaction was scored using a 10-point Likert scale. Five questions were related to the acquisition of self-management information, change in participants’ emotional states (such as anxiety and depression), improvement in their communication with the nurses, specific behavioral skills they had acquired, and enhancement of their social support. One question addressed the program’s necessity to help PLWH improve their self-management behaviors. Further, participants were asked open-ended questions regarding aspects of the app that were positive, and those that could be improved.

#### Disease and health-related characteristics

Participants responded to 11 health-related questions, including on the time of HIV diagnosis, source of health information, self-help group participation, smoking habits, alcohol consumption, exercise frequency, weekly breakfast schedule, night sleep duration, and sleep quality on a four-point Likert scale (very bad, bad, good, and very good).

Furthermore, they reported the date of their most recent blood test, and the viral load and CD4 + T cell counts were determined using a self-report questionnaire with three items. The responses were verified through the hospital’s electronic medical records after receiving the Institutional Review Board’s (IRB) approval and participants’ consent.

#### Demographic characteristics

Participants were requested to report their age, sex, marital status, education level, working status, type of residence, income, and sexual identity.

### Data collection

All participants were informed of the research purpose, content, and methods, and that their participation was voluntary, they could withdraw at any point, and their data would be kept confidential. All participants were requested to provide their informed consent online prior to participating in the study, and responded to the questionnaire only if they consented.

Both groups completed online self-report surveys using mobile phones. The data were collected thrice: immediately before the participants received their respective interventions; 4 weeks later, that is, immediately after the intervention ended; and 4 weeks after the intervention terminated, that is, 8 weeks after it began. During the second round, the intervention group was asked about how satisfied they were with the app. Gifticons ($10 per survey, total $30) were provided as an incentive for study participation.

### Statistical analysis

First, a descriptive analysis of the primary and secondary outcomes, disease and health-related characteristics, and demographic characteristics was performed. Then, a homogeneity test for dependent variables and characteristics of the intervention and control groups was conducted using the chi-square test, Fisher’s exact test, independent t-test, and Wilcoxon signed rank test. The comparison of primary and secondary outcome variables between the intervention and control group was conducted using the generalized estimating equation (GEE), a statistical method that can analyze repeated measures data; it helps assess non-normally distributed outcomes that frequently occur in actual clinical fields^32^. Here, covariates in the GEE analysis included the variables with significant differences between the groups in the homogeneity test results; further, the correlation structure was set as exchangeable. Additionally, the intention-to-treat analyses were performed. Finally, the differences between dropouts and participants were analyzed using the chi-square test, Fisher’s exact test, independent t-test, and Wilcoxon signed rank test. The data were analyzed using SPSS v25.0 (IBM Corp., Armonk, NY, USA) and Stata/SE 15.0 (StataCorp, TX, USA).

### Ethics approval

This study was approved by the IRB of the Yonsei University Health System (IRB Approval number: 4-2019-0884). The study was performed in accordance with the ethical standards laid down in the 1964 Declaration of Helsinki and its later amendments or comparable ethical standards.

### Consent to participate

The IRB of the Yonsei University Health System did not require the participants to provide written consent to evaluate the program’s effectiveness, given the study’s low risk and the fact that the intervention was provided through the Internet. All participants were informed of the research purpose, content, and methods, and that their participation was voluntary, they could withdraw at any point, and their data would be kept confidential. Before they responded to the survey, they were provided explanations of the study purpose and procedures. Moreover, they were able to respond to the questionnaire only upon agreeing to participate in the study.

## Results

### Participants’ baseline characteristics and homogeneity test results

**Figure 3:**
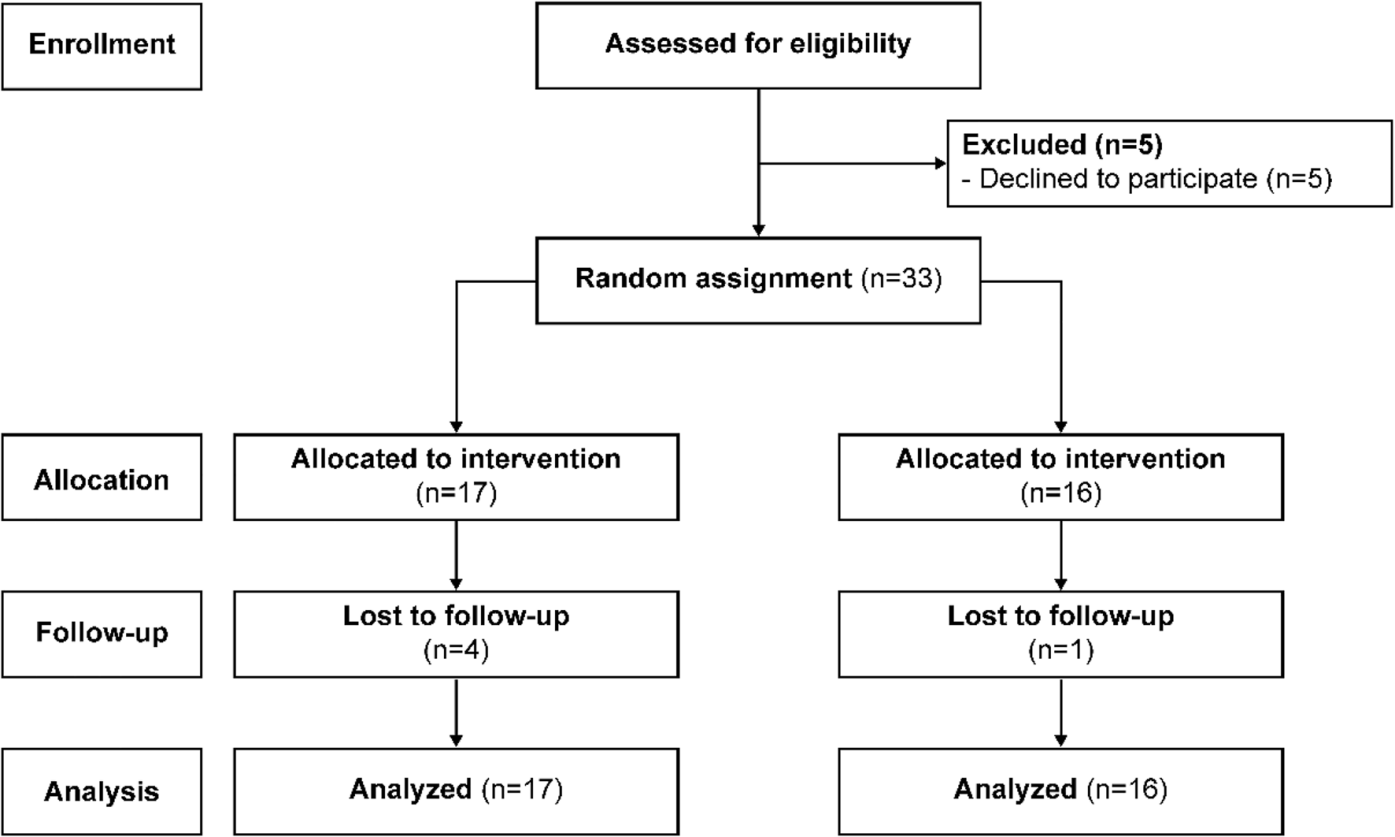
Participant flow diagram.

Of the 38 participants screened and 33 who were eligible, 17 were allocated to the intervention group and 16 to the control group (Fig. 3). Seventy-six percent (n = 13) of participants in the intervention group used the mobile application and completed all surveys. The participants’ characteristics and the homogeneity test results are shown in Supplementary Table S1. Sexual identity (χ^2^ = 6.114, *p* = 0.047), daily sleep hours (Z = 2.197, *p* = 0.028), and viral load (Z = 2.417, *p* = 0.016) were found to be significantly different between the two groups. In addition, self-efficacy for self-management showed a statistically significant difference between the two groups at baseline (t = 2.093, *p* = 0.046). The homogeneity test between the five dropouts and eligible participants demonstrated no statistically significant differences.

### Outcomes

**Figure 4:**
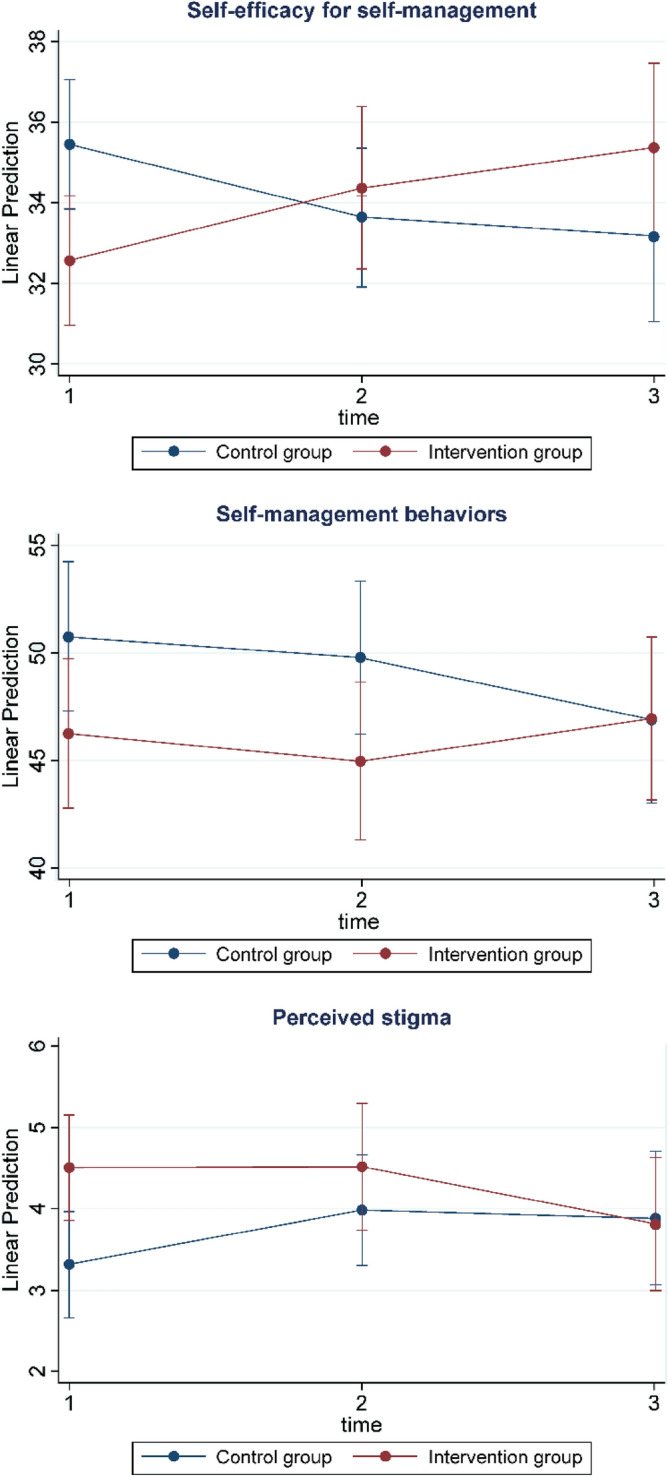
Changes in self-efficacy for self-management, self-management behaviors, and perceived stigma between the two groups.

Table 1 and Fig. 4 display the comparisons of the primary and secondary outcomes between the two groups. The analysis results indicated that the difference in self-efficacy for self-management (β = 2.292, *p* = 0.008), self-management behaviors (β = 2.660, *p* < 0.001), and perceived stigma (β = −0.704, *p* = 0.019) between the groups over time was statistically significant.

**Table 1:** Comparison of the changes in the scores of the self-management outcomes between the intervention and control groups (N = 33).

Outcome	Group	Estimated marginal means			Group effect	Time*Group interaction
		T0	T1	T2	β (95% CI)	β (95% CI)
**Primary outcomes**						
Self-efficacy for SM	Intervention	32.56	34.37	35.39	−3.425 (−7.110, 0.261)	2.292** (0.600, 3.985)
	Control	35.44	33.64	33.23		
SM behaviors	Intervention	46.25	44.93	46.92	−4.028 (−10.280, 2.223)	2.660*** (1.336, 3.984)
	Control	50.75	49.78	46.83		
Medication adherence	Intervention	92.50	99.06	99.46	−1.071 (−12.932, 10.790)	3.398 (−2.187, 8.984)
	Control	93.75	90.74	95.36		
**Secondary outcomes**						
Perceived health statu	Intervention	3.25	3.59	3.02	0.111 (−0.720, 0.942)	−0.102 (−0.496, 0.292)
	Control	3.31	3.29	3.37		
Depression	Intervention	8.56	5.50	7.67	1.603 (−4.358, 7.565)	−0.679 (−2.939, 1.580)
	Control	5.63	6.52	5.19		
Perceived stigma	Intervention	4.50	4.50	3.78	1.688* (0.279, 3.097)	−0.704* (−1.291, −0.116)
	Control	3.31	3.98	3.83		

^a^SM = Self-management.

^b^CI = Confidence Interval.

^c^T0 = baseline, T1 = second test (4 weeks), T2 = posttest (8 weeks).

^d^Sexual identity, daily sleep hours, viral load, and self-efficacy for self-management were included as covariates.

*p < .05, **p < .01, ***p < .001.

### Satisfaction with the program

The results of satisfaction with the program are presented in Table 2. The highest satisfaction scores were for acquiring behavioral skills and self-management information, at 7.09 (± 2.587), and 6.64 (± 2.976), respectively. However, the changing emotional states satisfaction score was the lowest at 4.83 (± 2.637) points. The score for necessity of the intervention for helping PLWH to improve self-management was 6.91 (± 2.587) points. Box 1 displays the participants’ qualitative feedback regarding the program.

**Table 2:** Satisfaction with the program.

Variables	Mean ± SD
Acquiring behavioral skills	7.09 ± 2.587
Acquiring self-management information	6.64 ± 2.976
Improving communication with nurses	5.45 ± 2.659
Improving social support	5.09 ± 3.534
Changing emotional states (such as anxiety and depression)	4.82 ± 2.639
Total	6.00 ± 2.888

**Box 1: Participants’ comments based on their experience of the intervention**

1. Positive and helpful aspects of the program
  Convenience and easy accessibility of the necessary information
  Useful health information that I did not know
  Helps to consider and practice health behaviors
  Good for checking and monitoring my health practices
  Anonymity of the program
  The function to ask a nurse through the mobile app seemed to be very useful because it is difficult for the people living with HIV to disclose their infection status and participate in self-help groups
2. Program areas requiring improvement
  Providing the program for patients with early diagnosis
  Introducing variety in case management and contents
  Dividing and managing the HIV-related and general health information
  Modifying the detailed functions in a community post to increase its convenience
  Introducing automatic login function
  Emphasizing that personal information is safely protected

## Discussion

This study aimed to evaluate the effectiveness of a self-management program using a mobile-based app called the “Health Manager,” to improve the self-management outcomes of PLWH during the COVID-19 pandemic. Statistically significant differences were found between the intervention and control groups for self-efficacy for self-management, self-management behaviors, and perceived stigma of HIV.

Self-efficacy for self-management significantly improved over time in the intervention group compared to the control group. Damush et al. reported participants’ improvements in specific self-efficacy for pain management through the provision of interventions including patient education, goal-setting, self-monitoring, and problem-solving^33^. In the current study, the provision of effective self-management skills through the app functions of “Health goals,” “Health diary,” and “Alarm” was considered to have led to the participants’ successfully achieving planned goals. Self-efficacy for self-management significantly affects quality of life among PLWH^24^; Park et al. reported its importance in explaining health promotion behaviors, and the need to enhance the same^19^. Therefore, the program proposed in this study could be used as an intervention to improve the self-efficacy for self-management among PLWH.

The intervention group’s self-management behaviors significantly improved over time as compared to the control group. This may have occurred as the program provided self-management information, motivation, and behavioral skills to promote changes in self-management behavior based on the IMB model^23^. In addition, while previous studies provided app-based interventions focusing on medication adherence^34,35^, this study included various self-management elements such as medication adherence, smoking, alcohol consumption, and sex life in the program. In previous studies, self-management behaviors such as sexual behavior and health practices along with medication adherence were suggested as elements for improving self-management among PLWH^26,36^. Thus, to improve the self-management behaviors of PLWH, it is necessary to consider interventions that can improve information, motivation, and behavioral skills for various self-management elements.

Perceived stigma significantly decreased over time in the intervention group as compared to the control group. Recently, a self-management intervention study of PLWH using technology included stigma as a secondary outcome^37^. Another research attempted to improve the stigma against PLWH through a program that utilized multimedia to provide content about stigma and depression^38^. PLWH in Korea have experienced high levels of internalized stigma or self-stigmatization; they found it challenging to tell others about their HIV infection status due to stigma and discrimination^39^. In this study, the participants’ average stigma score at baseline was 3.85 points, higher than the 2.4 points reported in a study involving American adults living with HIV^40^. Additionally, one participant reported difficulty in participating in the community’s self-help group for PLWH. Thus, providing a space for PLWH to disclose their HIV infection status and interact anonymously can reduce their perceived stigma.

The intervention group showed an improvement in medication adherence over time; however, there was no statistically significant difference from the control group. In this program, an attempt was made to improve medication adherence by using functions such as video educational materials and medication reminders on medication adherence. The average medication adherence score of participants in this study was 93.03 points, higher than the 89.5 points reported in a study involving African American PLWH^41^, and it may be difficult to identify changes in participants who already have high medication adherence. In addition, each participant was expected to have their own strategy for taking medication, considering the average period after their HIV infection diagnosis (8.47 ± 6.246 and 7.44 ± 5.966 years for the intervention and control groups, respectively). Nevertheless, newly diagnosed PLWH experience difficulty in medication adherence because of psychological distress and issues with taking medications^8^. As reported by the participants, providing this program to PLWH during the early stages of their diagnosis can help improve medication adherence. Therefore, it is necessary to provide this program to PLWH at the initial stage of diagnosis or to participants who have difficulties in medication adherence in the future.

Although depression decreased over time in the intervention group, the difference between the intervention and control groups was not statistically significant. In addition, the satisfaction level in the aspect of emotional change was the lowest, at 4.82. In this study, the researcher attempted to reduce depression by providing educational materials through health information bulletin boards and the operating community as well as through counseling with nurses; however, a more systematic approach is needed. In a previous study reporting reduced depression of PLWH with an app-based program, reminders and short articles about stress reduction were provided three to five times a week for 3 months^42^. In another study, a personalized self-management strategy tailored to the participants’ reported symptom experience was provided using 3–27 s video training materials^17^. In this study, the intervention period was relatively short with insufficient content focusing on depression; thus, including additional materials for improving depression and personalized strategies in the app is vital to lessening depression.

The participants of this study exhibited low satisfaction with the improvement of communication with nurses (5.90 points) and social support (5.45 points). The factors influencing the usability of apps include both usefulness of its information, and its easy manipulation, playfulness^43^, and trustworthiness^44^. The app developed in this study did not include elements that could build trust with the users, elements that could increase interest such as through virtual reality^45^ or gamified team-building^46^, and the user experience design process. In addition, another study on self-management app usability among PLWH found that the usability of the app sections differed according to the participants’ HIV infection stage^47^. For example, PLWH in the early disease stage used educational information more frequently, and PLWH in the later disease stage used reminders more often^47^. In future research, it may be valuable to include additional elements to improve usability of the app-based self-management program and provide customized functions by considering the disease stage of PLWH.

This study has some limitations. First, the sample size was small and participants were recruited from a single hospital, which limits the generalizability of this study. Second, this research employed random assignment; however, the characteristics of the intervention and control groups were not homogeneous. Therefore, the researcher presented the statistical analysis results adjusted for the covariate variables. Third, the nature of the intervention prevented double blinding; hence, threats to validity such as experimenter expectancies (e.g., Rosenthal effect) cannot be excluded. Fourth, since the intervention was provided for 4 weeks, and outcomes were measured up to 8 weeks, its long-term impact could not be confirmed. Lastly, the self-efficacy in self-management and perceived stigma scales were not examined for validity and reliability after their translation into Korean.

## Conclusions

This study was conducted to evaluate the effects of a self-management program using a mobile app on self-management outcomes for PLWH in Korea during the COVID-19 pandemic. This intervention significantly improved self-efficacy for self-management, and self-management behaviors, and reduced the perceived stigma of PLWH in Korea. The results indicated satisfaction with this program through the high score measured regarding acquiring behavioral skills and information, supported by the participants’ qualitative comments. Therefore, HCPs in the field could consider using similar interventions with apps to enhance self-efficacy for self-management, self-management behaviors, and perceived stigma. This research can be employed in various fields to counsel and educate PLWH regarding self-management. Additionally, it can provide a basis for research to develop and examine mobile app-based self-management interventions during crises such as pandemics.

## Supplementary Information

Supplementary Information.

## Data Availability

The data that support the findings of this study are available on request from the corresponding author, G.S.K. The data are not publicly available due to their containing information that could compromise the privacy of research participants.
