# Supplementary material for: Developing an app-based self-management program for people living with HIV: a randomized controlled pilot study during the COVID-19 pandemic
Source: Sci Rep. 2022 Nov 12;12:19401. doi: 10.1038/s41598-022-19238-w (PMC9653395; doi:10.1038/s41598-022-19238-w)
Supplement: Supplementary file 1 — Supplementary Information. [file 41598_2022_19238_MOESM1_ESM.docx]

## Supplementary Information

Table S1. Participants’ baseline characteristics and homogeneity test results (N=33).

| Variable | Category | Overall | Intervention group (n=17) | Control group (n=16) | t/Z/ **χ^2^** |
| --- | --- | --- | --- | --- | --- |
|  |  | N(%) | N(%) | N(%) |  |
|  |  | Mean±SD | Mean±SD | Mean±SD |  |
| **Demographic** | |  |  |  |  |
| Age (in years) | | 42.03±10.646 | 41.82±9.939 | 42.25±11.676 | 0.113 |
| Sex | Male | 33(100.0) | 17(100.0) | 16(100.0) | - |
| Education  level | ≤High school | 9(27.3) | 2(11.8) | 7(43.8) | 4.251 |
|  | ≥college | 24(72.7) | 15(88.2) | 9(56.3) |  |
| Economic status | High | 3(9.1) | 1(5.9) | 2(12.5) | 0.593 |
|  | Middle | 21(63.6) | 11(64.7) | 10(62.5) |  |
|  | Low | 9(27.3) | 5(29.4) | 4(25.0) |  |
| Marital status | Never married | 28(84.8) | 14(82.4) | 14(87.5) | 2.014 |
|  | Married | 3(9.1) | 1(5.9) | 2(12.5) |  |
|  | Others^c^ | 2(6.1) | 2(11.8) | 0(0.0) |  |
| Residence | Alone | 16(48.5) | 8(47.1) | 8(50.0) | 1.608 |
|  | With family | 13(39.4) | 8(47.1) | 5(31.3) |  |
|  | With friend | 4(12.1) | 1(5.9) | 3(18.8) |  |
| Working | Yes | 20(60.6) | 9(52.9) | 11(68.8) | 0.863 |
|  | No | 13(39.4) | 8(47.1) | 5(31.3) |  |
| Sexual identity | Homosexual | 21(63.6) | 10(58.8) | 11(68.8) | 6.114* |
|  | Heterosexual | 7(21.2) | 2(11.8) | 5(31.3) |  |
|  | Others^d^ | 5(15.2) | 5(29.4) | 0(0.0) |  |
| Information source | Internet | 11(33.3) | 6(35.3) | 5(31.3) | 2.404 |
|  | HCPs^a^ | 17(51.5) | 10(58.8) | 7(43.8) |  |
|  | PLWH^b^ community | 2(6.1) | 0(0.0) | 2(12.5) |  |
|  | TV/radio | 3(9.1) | 1(5.9) | 2(12.6) |  |
| **Disease-related** | |  |  |  |  |
| Years after diagnosis | | 7.97±6.039 | 8.47±6.246 | 7.44±5.966 | 0.482 |
| Self-help group  participation | Yes | 2(6.1) | 0(0.0) | 2(12.5) | 2.262 |
|  | No | 31(93.9) | 17(0.0) | 14(87.5) |  |
| **Health-related** | |  |  |  |  |
| Smoking | Yes | 13(39.4) | 7(41.2) | 6(37.5) | 0.047 |
|  | No | 20(60.6) | 10(58.8) | 10(62.5) |  |
| Alcohol consumption | ≥1 per month | 17(51.5) | 7(41.2) | 10(62.5) | 1.861 |
|  | <1 per month | 7(21.2) | 5(29.4) | 2(12.5) |  |
|  | None | 9(27.3) | 5(29.4) | 4(25.0) |  |
| Exercise | <3 per week | 19(57.6) | 10(58.8) | 9(56.3) | 0.022 |
|  | ≥3 per week | 14(42.4) | 7(41.2) | 7(43.8) |  |
| No. of breakfast per week | | 3.79±2.408 | 4.12±2.395 | 3.44±2.449 | 0.800 |
| Daily sleep hours | | 6.76±1.306 | 6.41±1.502 | 7.13±0.975 | 2.197* |
| Sleep quality | Good | 18(54.5) | 9(52.9) | 9(56.3) | 0.036 |
|  | Bad | 15(45.5) | 8(47.1) | 7(43.8) |  |
| Viral load (copies/ml) | | 11.59  ±22.218 | 4.92  ±19.055 | 17.84  ±23.711 | 2.417* |
| CD4+ T cell counts (per μl) | | 678.55  ±262.316 | 769.67  ±261.522 | 593.13  ±240.207 | 1.937 |
| **Primary outcomes** | |  |  |  |  |
| Self-efficacy for self-management | | 34.03±3.996 | 32.71±4.647 | 35.44±2.632 | 2.093* |
| Self-management behavior | | 48.39±7.717 | 46.18±9.561 | 50.75±4.250 | 1.756 |
| Medication adherence | | 93.03±8.095 | 92.35±9.701 | 93.75±6.191 | 0.040 |
| **Secondary outcomes** | |  |  |  |  |
| Perceived health status | | 3.27±0.761 | 3.24±0.752 | 3.31±0.793 | 0.567 |
| Depression | | 7.33±6.288 | 8.94±7.701 | 5.63±3.879 | 1.123 |
| Perceived stigma | | 3.85±1.603 | 4.35±1.618 | 3.31±1.448 | 1.857 |

^a^HCPs=Health care providers

^b^PLWH=People living with HIV

^c^Includes divorced (n=1) and separated (n=1) individuals

^d^Includes bisexuals (n=3) and asexuals (n=2)

* *p*<.05
